# Visceral Leishmaniasis Outbreak in South Sudan 2009–2012: Epidemiological Assessment and Impact of a Multisectoral Response

**DOI:** 10.1371/journal.pntd.0002720

**Published:** 2014-03-27

**Authors:** Abdinasir Abubakar, José Antonio Ruiz-Postigo, Jane Pita, Mounir Lado, Riadh Ben-Ismail, Daniel Argaw, Jorge Alvar

**Affiliations:** 1 World Health Organization, Juba, South Sudan; 2 World Health Organization, Cairo, Egypt; 3 Ministry of Health, Juba, South Sudan; 4 World Health Organization, Geneva, Switzerland; Institute of Tropical Medicine, Belgium

## Introduction

The humanitarian situation in South Sudan is dire, with over two million returnees since 2005 and another 300,000 expected to return by the end of 2013. In 2012, 170,000 refugees settled in five camps in Unity and Upper Nile states, endemic areas of visceral leishmaniasis (VL) (Office for the Coordination of Humanitarian Affairs Sudan Humanitarian Update 1st Quarter 2012).

VL in South Sudan is endemic in four states, namely Upper Nile, Unity, Jonglei, and Eastern Equatoria, where 2.7 million people in 28 counties are considered to be at risk.

It is an anthroponosis caused by *Leishmania donovani*, and the vectors are *Phlebotomus orientalis* and *P. martini.* South Sudan is suffering from recurrent epidemics in areas previously considered to be nonendemic [1].

VL in South Sudan was first described in a child from Bahr-el-Ghazal in 1904 [2]. Since then, outbreaks have been reported in several different areas, namely Jonglei state in the 1930s and 60s [3], [4], the former Blue Nile province in the 50s [5], and Unity state (formerly Western Upper Nile) in the 80s, until by the 90s it was claimed that almost one-third of the population had been affected between 1984 and 1994 [6]–[8]. In 1994 and in 2002 there were two further outbreaks in northern Jonglei and Eastern Upper Nile states, resulting in 17,000 cases reported. Since 2002, the number of cases reported has progressively decreased to reach 582 in 2008. From 2004 to 2008, an average of 1,756 cases were reported annually, although the actual number of cases was estimated to be between 7,400 and 14,200 cases [9].

Until 2004, VL treatment services were provided almost exclusively by Médecins Sans Frontières-Holland (MSF-H), after which time part of the VL-treatment activities were handed over to the Southern Sudan Secretariat of Health. In addition to this, the World Health Organization (WHO) supported eight health facilities run by nongovernmental organizations (NGOs), and in 2009, that network was expanded to 12.

Another VL outbreak was declared in 2009 and it is still ongoing. This publication provides a detailed report on the epidemiology of and the response to this outbreak.

## Methods

Retrospective data collection and analysis was based on field records from 25 health facilities, line listing of cases, and standardized reporting forms for all health facilities. Records included data on the type of disease (primary, relapse, or post kala-azar dermal leishmaniasis), age, gender, and treatment outcome. Statistical tests for data analysis and comparison of percentages were done using the Chi-squared test.

## Results

In September 2009, an increased number of new VL cases was reported in Malakal Teaching Hospital (Upper Nile state) and Old Fangak Primary Health Care Centre (PHCC) (Jonglei state). In subsequent months, most NGOs operating in the area recorded a similar trend.

**Figure 1:**
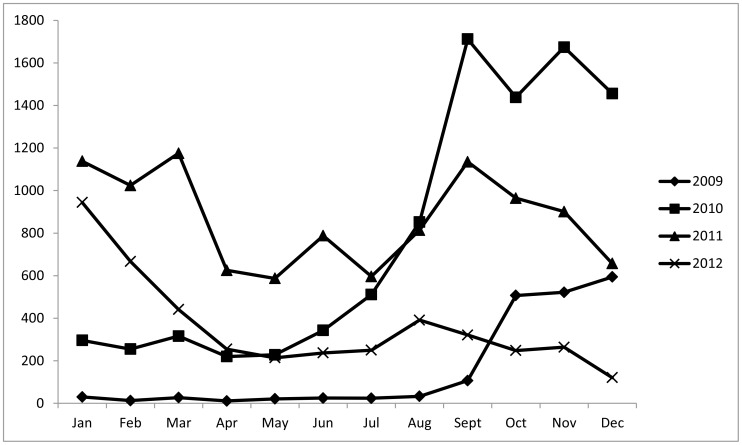
Number of new visceral leishmaniasis cases per month in South Sudan, 2009–2012.

**Figure 2:**
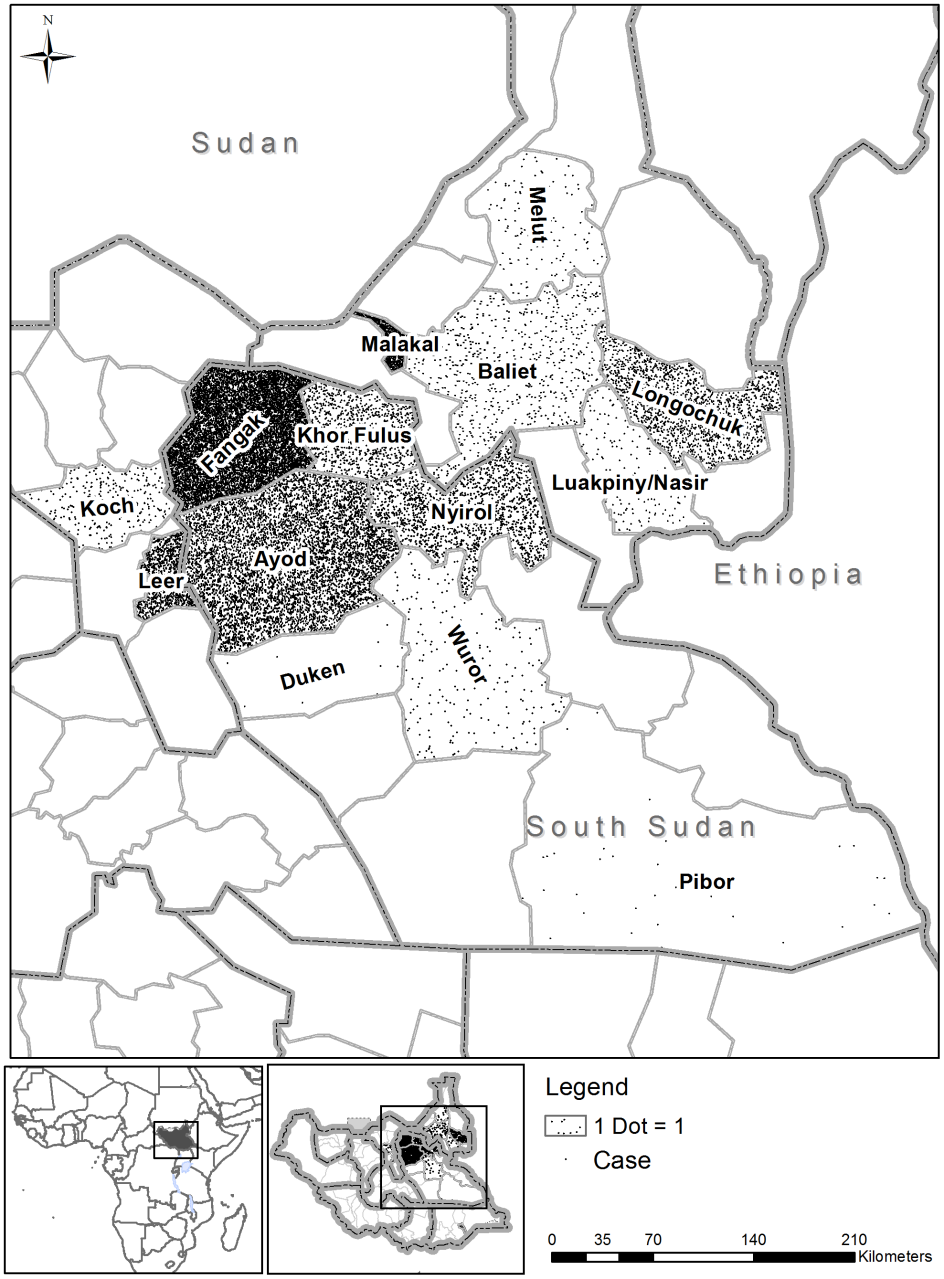
Geographical distribution of visceral leishmaniasis cases by county in South Sudan, 2009–2012.

From September 2009 until December 2012, over 76,000 VL suspects were screened and 28,328 new cases of VL were reported (Figure 1). Treatment was provided in 25 centres in 16 counties (Figure 2).

Of these cases, 90% (27,050/28,328) were primary VL cases; 72% (20,382/28,328) were patients less than 17 years of age, and 52% (14,721/28,328) were males.

The monthly distribution of cases reflected the general trend observed in South Sudan, with fewer cases during the transmission season (April to June) and a peak during the dry season, starting progressively in September [10].

Nutritional status was assessed in Jiech and Old Fangak, where 54% (1,907/3,542) and 75% (240/320) of patients were either severely or moderately malnourished, as per the definitions from national guidelines [11].

The overall case fatality rate (CFR) observed was 3% (855/28,328). The CFR decreased progressively from 6% (115/1,914) in 2009 to 4.1% (393/9,695) in 2010 (p<0.001) and from 2.5% (293/11,888) in 2011 to 1.5% (77/5,015) in 2012 (p<0.001).

The majority of cases were reported in five health centres, namely Old Fangak PHCC (35%, 9,772/28,328), Jiech Primary Health Care Unit (PHCU) (12%), Ayod PHCC (8%), Malakal hospital (7%), and Lankien PHCC (7%), and came from three counties: 35% (9,772/28,328) from Fangak, 24% (6,830/28,328) from Ayod, and 7% (2,116/28,328) from Nyirol, within Jonglei state.

Relapses represented 1.6% (170/10,798) of cases in 2010 and 8.6% (432/5,015) of cases in 2012. Post kala-azar dermal leishmaniasis (PKDL) was noted in 10.4% (1,118/10,798) of patients in 2010 and 4.6% (230/5,015) of patients in 2012.

### Outbreak response and intervention strategy

The response to the outbreak was organized as a multisector approach. Access to treatment was improved by decentralizing case management. The number of treatment centres was increased from eight in 2008 to 25 by December 2011 (23 run by NGOs and two by the Ministry of Health [MoH]). In some areas, however, people reached the nearest centre after 2–3 days of walking. The mortality rate of patients who were required to walk long distances to access care is unknown.

Task forces were set up by the MoH in Juba and Malakal to strengthen coordination between stakeholders. Data collected through new standardized forms by all implementing partners were analysed and shared on a weekly and monthly basis.

WHO trained 320 health personnel in case management and epidemiological surveillance. Standard operating procedures and guidelines for case management and nutritional support were developed.

MSF-H and WHO supplied diagnostic tests and medicines. Serological diagnosis was made using the rK39 rapid immunochromatographic test on individuals suspected to have primary VL on clinical assessment, with fever >2 weeks and splenomegaly or wasting, after malaria and previous VL had been ruled out. In four centres, the direct agglutination test (DAT) was also available for those suspected to have VL but with a rK39-negative result. Lymph node aspirates were performed for parasitological assessment. Only one centre was able to perform spleen aspirates. In November 2010, sodium stibogluconate (SSG) monotherapy, 20 mg Sb5+/kg/day over 30 days, was replaced with SSG plus paromomycin (PM) combination therapy, 20 mg Sb5+/kg/day plus PM 15 mg/kg/day over 17 days, as the national first-line treatment and according to new WHO recommendations [12]. Liposomal amphotericin B was used for severe cases, pregnant women, and HIV-VL co-infected patients in facilities with cold chain. UNICEF (United Nations Children's Fund) and WFP (World Food Programme) supplied nutritional supplements to all treatment centres.

Lack of precise entomological information made it impossible to implement proper vector control measures. However, in an attempt to decrease vector-human contact, 2,309,991 long-lasting impregnated bed nets (LLINs) were distributed to all VL-endemic areas and 45,000 LLINs to VL treatment centres. Health partners engaged in health education campaigns focusing on proper use of LLINs, nutrition, and VL health services available.

### Challenges

The ongoing insecurity in northern Jonglei and Upper Nile played a major part in preventing patients from seeking medical care for several months between 2010 and 2011. Logistics were very challenging and costly. There are no roads in this region, and only Malakal has an all-weather tarmac airport; all other landing strips are operable only during the dry season, through chartered flights. Most VL treatment facilities were inaccessible during the rainy season (May to October), and limited storage capacity meant deliveries were required on a monthly basis. Lack of cold chain in most treatment facilities prevented the wide introduction of Liposomal amphotericin B. In most centres there was a limited number of health staff and/or insufficient trained personnel due to high turnover.

## Discussion

VL in South Sudan occurs in epidemic cycles; therefore, a new outbreak was already a concern in 2008 when the case load was still low [13]. Several factors are believed to have exacerbated the scale of this recent outbreak, including massive population movement, malnutrition, and diminished herd immunity. Population movement dramatically influences the spread of VL, which here was most prominent in northern Jonglei counties, the area where over 60% of cases occurred.

The response to the outbreak was made through a concerted effort between the MoH, United Nations agencies, and NGOs. Despite the challenges faced, the approach appeared relatively successful, and the decrease observed in the CFR compared to former outbreaks could be attributed to improved access to treatment and standards of care, including the use of SSG/PM. This was the first time in the last 20 years that the annual CFR in South Sudan was brought to below 4%. Interpretation of the CFR, however, needs to be made with caution due to three main factors: (i) If people do not strictly adhere to the clinical case definition for suspected VL prior to serological testing with rK39, the specificity of the rK39 test decreases considerably, and the proportion of asymptomatic infections identified is significant. This proportion of “false-positive” VL cases increases during the declining phase of the epidemic, when a large proportion of the population has been infected, but have not developed clinical VL. (ii) During the declining phase of the epidemic, the proportion of true VL cases among clinical suspects decreases, and rK39 positivity rates decrease (to much lower than the 50–60% positivity rate expected during the height of the epidemic), which results in a decreasing positive predictive value of the rK39 test. (iii) Incompleteness of field registers did not allow exhaustive data on the treatment outcome in all health facilities. That may have biased the data analysis and limits the extent to which the actual CFR can be ascertained.

Although there was no exhaustive patient follow-up, the increased proportion of relapses among the patients admitted over time could be also attributed to the use of SSG/PM as first-line treatment and the lower case fatality rate observed [14]. A similar trend has been observed in health facilities which had already implemented that treatment protocol before the outbreak. However, the fact that in the declining phase of the epidemic the number of primary VL admissions decreases results in an increasing proportion of relapses among the total admissions. This might explain the observed increased retreatment rate.

Despite the improved outbreak response, the recurrent pattern of VL outbreaks in South Sudan and the near impossibility of breaking this cycle remain major public health concerns. The number of people who died because they could not reach a treatment centre is unknown. The true incidence of VL in South Sudan is difficult to assess because of the poor health infrastructure, limited accessibility and availability of health services, and weak surveillance system [15].

With large numbers of new returnees settling in South Sudan and continued instability, it is expected that VL will continue to wreak havoc on an extremely vulnerable population.
